# The Relationship between Technology Addiction and Attitude toward Reading: An Investigation on Pre-Service Teachers

**DOI:** 10.3390/bs13090775

**Published:** 2023-09-16

**Authors:** Fatma Gerez Taşgın, Adnan Taşgın

**Affiliations:** 1 Department of Turkish Language Education, Atatürk University, Erzurum 25240, Turkey; fatmatasgin@atauni.edu.tr; 2 Department of Educational Science, Atatürk University, Erzurum 25240, Turkey

**Keywords:** attitude, reading, technology addiction, pre-service teachers

## Abstract

In this study, we aimed to examine pre-service teachers’ attitudes toward reading books and their technology addictions. We used a correlational survey model. The sample of this study consisted of a Turkish pre-service teachers group studying at the Department of Turkish Education in the education faculty of a state university in Turkey. Our research used the “Attitude Scale Towards Reading” and “Technology Addiction Scale” as data collection tools. As a result of the study, we determined that the attitudes towards reading of pre-service teachers were positive. Furthermore, there was a significant difference in favor of female pre-service teachers in the sub-dimensions of book-reading love, reluctance to read, and stress. The significant difference in the online game-playing sub-dimension of technology addiction favored male pre-service teachers. On the other hand, we found a negative meaningful relationship between pre-service teachers’ technology addiction and their attitudes toward reading.

## 1. Introduction

The rapid progress of information and technology has enabled technological products to permeate human life swiftly. At the same time, information and technology have become indispensable aspects of our lives. With these technological advancements, human life has become notably easier across various domains, and technical products have demonstrated their influence in nearly every facet of human existence. Due to the shifts in recent years, human needs have become more diverse, and the concept of socialization has undergone transformations. Concurrently, technological advancements have empowered individuals to communicate via social networks [1]. The evolution of technology has brought about numerous conveniences to human life. Alongside these advantages, it has also given rise to psychological challenges affecting individuals [2,3,4]. The concept that emerges due to the unconscious use of technology is called technology addiction.

Technology addiction is defined as “excessive use of technology, inability to satisfy the desire to use technology, neglecting daily activities due to excessive use of technology, weakening of social relations due to excessive use of technology, employing technology as an escape mechanism from negative emotions and life stress, struggling to reduce and give up technology use, being nervous and irritable when using technology is not possible, and providing false information about the extent and duration of using technology” [5,6,7].

Technology causes a variety of worries, from anxiety disorders to panic attacks [8,9]. Parents fear computer anxiety and the dangers of the internet, not only for themselves but also for their children and grandchildren [10]. Parents with obsessive–compulsive disorder cannot avoid becoming addicted because they cannot control their impulses when they are too busy with digital technologies such as e-mail, shopping, or video games [11]. The overuse of technology creates problems with tolerance, conflict, mood swings, and withdrawal, which show some signs of behavioral addiction [5,12]. Technology addiction can endanger the user’s social life, impair emotional functioning, negatively affect school, family, and work life, and the person’s social environment [13,14].

Upon reviewing studies concerning technology addiction, it becomes evident that there are adverse effects on students. Widyanto and Griffiths [15] discovered that individuals who engage in virtual environments experience lower self-esteem. They explained the reason for low self-esteem is that individuals can escape from real-life problems more easily in the virtual world. Individuals with low self-esteem in real life become more dependent on the virtual environment. High and Caplan [16] found that individuals mostly use technologies and virtual communication networks to avoid negative criticism in real life and suppress anxiety. Some studies conclude that smartphones cause negative effects on the psychology of children and young people [17]. Doğan and İlçin-Tosun [18] reported that there was a relationship between high school students’ social networking sites and problematic smartphone use and their social anxiety levels. Furthermore, these researchers concluded that an escalation in problematic smartphone use corresponded to an increase in the social anxiety levels of the students. On the other hand, Elmas et al. [19] found that the duration of medical school students’ internet presence negatively affects their school success. Lee et al. [20] stated that women used smartphones more frequently than men. Also, their study reported a relationship between psychological characteristics and smartphone use. The results show that smartphone use positively affects technostress. Hawi and Samaha [21] found that students with high addiction also had high anxiety. In addition, these researchers concluded that students with high anxiety are more likely to have problems in family relationships. Karaman and Kurtoğlu [22] found a significant difference between pre-service teachers’ genders and internet addiction. They concluded that this difference was in favor of male pre-service teachers. Chen et al. [23] concluded that mobile-phone-addicted individuals are more sensitive to negative emotions and spend more time and money on mobile phones. Filiz et al. [24] found a moderately positive and significant relationship between students’ internet addiction and the purpose of using social networks.

The increase in the mobile addiction of individuals [23] and the decrease in the quality of time give clues about the decrease in the time allocated to reading books. One reason for the decline in book reading is technology addiction [25].

Reading makes a significant contribution to individuals’ comprehension skills. Individuals who do not take the time to read or see reading only as a recreational activity may find it difficult to understand themselves and their surroundings over time. Individuals with low comprehension skills may stop thinking about what they find difficult to comprehend. They may eventually isolate themselves from the ability to think, which is an important language component. One of the essential concepts related to reading is developing reading habits. Reading habit is defined as an action that forms the basis of lifelong learning [26], that an individual performs regularly, critically, and continuously by perceiving the act of reading as a source of need and pleasure [27].

In the literature, various studies have focused on the attitudes toward the habit of reading. Some of these studies concluded that reading habits showed a statistically significant difference in favor of female students according to gender variable [28,29,30,31]. In contrast, others stated there was no differentiation in the class variable [31,32]. From the previous studies about the attitude toward reading, Çetinkaya-Edizer [33] found that pre-service teachers’ metacognitive reading strategies positively related to their attitudes toward reading habits. Moreover, Granado [34] concluded that pre-service teachers were not interested in reading, did not turn to various reading texts, and did not value books much. Furthermore, Demir [35] found a significant positive relationship between college students’ reading proficiencies, consciousness, and reading habits. Ürün-Karahan [36], on the other hand, found that pre-service teachers’ views on reading attitudes were positive. These differences in the studies may be due to both the sample and the cultural approach to the teaching profession.

Reading significantly improves the comprehension skills of individuals. Failure to develop an individual’s reading skills leads to a lack of understanding of oneself and one’s surroundings over time and difficulties in generating ideas and thinking effectively. The reading habit is the act of reading consistently, critically, and continuously throughout life. Having the habit of reading forms the basis of lifelong learning. Technology addiction or mobile addiction will cause a decrease in reading time. At the same time, these addictions will negatively affect individuals’ comprehension skills, language skills, and ability to generate ideas in general.

The studies conducted in the literature show that pre-service teachers who read more books have lower technology addiction [37]. In addition, there are also results that as the rate of reading books by pre-service teachers decreases, their interest in technology increases and negatively affects health [37,38]. In this context, teachers raising future generations should also have reading habits because a teacher should show exemplary role-model behaviors to their students. On the other hand, as another important variable of this research, a teacher should not be addicted to technology. In this context, it is important to examine the relationship between technology addiction and the reading habits of pre-service teachers who will be exemplary role models for future generations.

This study examined the relationship between pre-service teachers’ attitudes toward reading books and their technology addictions. For this purpose, the hypotheses of the research were as follows:

- According to the gender variable, pre-service teachers’ attitudes toward reading and technology addiction differ in favor of female students;
- There is a significant negative relationship between pre-service teachers’ attitudes toward reading and their technology addiction.

## 2. Materials and Methods

### 2.1. Research Design

The model of this research was a correlational survey, one of the quantitative research types. In relational research designs, researchers usually apply the Pearson correlation test to measure and describe the degree of relationship between two or more variables. In this type of design, researchers do not attempt to control or manipulate variables as carried out in an experimental design. Instead, they correlate two or more scores for each individual using the correlation method [39].

### 2.2. Population and Sample

The population of this research consisted of Turkish pre-service teachers studying in the Department of Turkish Education at a state university in Turkey. We selected the sample of the study according to the simple random sampling method. There were 212 Turkish pre-service teachers in the sample. Of the pre-service teachers, 146 (68.9%) were female and 66 (31.1%) were male. Of the pre-service teachers, 26 (12.3%) were in the 1st grade, 80 (37.7%) in the 2nd grade, 46 (21.7%) in the 3rd grade, and 60 (28.3%) in the 4th grade.

### 2.3. Data Collection

We collected our research data using two data collection tools in the study.

“Technology Addiction Scale”: The scale developed by Aydın [40] consists of four sub-dimensions and 24 items. The scale was developed on a 5-point Likert scale. Some of the scale items are: “I play online games to forget about real-life problems.”, “I neglect my studies to surf websites.”, and “I want to increase the time I spend on social networks even though I have more important things to do.”. The Cronbach’s alpha coefficient calculated by the developer of the scale was 0.95. The Cronbach alpha reliability coefficients we calculated for our study were 0.748, 0.752, 0.856, and 0.844 for the four dimensions. It was 0.910 for the whole scale.

“Attitude Scale Towards Reading Books”: The scale developed by Doğan and Çermik [41] consists of 4 factors and 36 items. The scale was developed on a 5-point Likert scale. Some of the scale items include: “Reading is one of my favorite things.”, “I don’t like reading because I can’t focus on it.”, “Reading books is boring.”, and “I believe that reading develops my imagination.”. The Cronbach’s alpha coefficient calculated by the researchers who developed the scale was 0.95. The Cronbach alpha reliability coefficients we calculated for our study were 0.905, 0.807, 0.712, and 0.897 for the four dimensions. It was 0.880 for the whole scale.

### 2.4. Data Analysis

MANOVA analysis was used to analyze the data. Before performing MANOVA analysis, we tested the MANOVA assumptions, which were the independence of the observations, the normality values, and the homogeneity of the covariances. We observed a moderate correlation among the sub-dimensions of the scales. In this case, it showed that the multicollinearity assumption was met. We used the α = 0.05 significance level for all statistical tests. We conducted a Pearson correlation analysis to determine the relationship between technology addiction and attitude toward reading.

## 3. Results

The descriptive statistics of pre-service teachers’ attitudes toward reading books according to gender variables are shown in Table 1.

MANOVA analysis results on whether pre-service teachers’ attitudes toward reading books differ according to gender are shown in Table 2.

First, we checked the assumptions of multivariate analysis of variance (MANOVA). We found that the homogeneity assumption of the spread matrix (Box’s M) was met. (F_(10–77,841.308)_ = 1.797, Sig. = 0.055). According to the results of Wilks’ Lambda test (Table 2), it was concluded that the linear combinations of the sub-dimensions of the pre-service teachers’ book reading attitude scale differed significantly according to the gender variable (Wilk’s Λ = 0.903, F_(4,207)_ = 5549, Sig. = 0.000, ƞ^2^ = 0.097). Cohen [42] classified the effect size values as ƞ^2^ = 0.01 “small”, ƞ^2^ = 0.06 “moderate”, and ƞ^2^ = 0.14 and above as “large” effect. The effect size value calculated as 0.097 was determined to be above the medium level. The analysis results regarding which dependent variables had significant differences are shown in Table 3.

According to Table 3, we found a significant difference in the love of reading, reluctance to read, and stress sub-dimensions (F_love of reading (1–210)_ = 18,868, Sig. = 0.000, ƞ^2^ = 0.082; F_reluctance and stress in reading (1–210)_= 7011, Sig. = 0.009, ƞ^2^ = 0.032). After conducting this analysis, the effect size values calculated between the gender variable and the two sub-dimensions with a significant difference were close to the medium level according to the classification of Cohen [42]. By examining the mean scores, it was understood that female pre-service teachers ($\bar{X}$ = 45.23) loved to read more books than male pre-service teachers ($\bar{X}$ = 39.17) and it was also found that female pre-service teachers ($\bar{X}$ = 38.87) were more reluctant and stressed than male pre-service teachers ($\bar{X}$ = 37.40) in the reluctance and stress subscale of reading books. The descriptive statistics about the technology addiction of pre-service teachers according to gender variables are shown in Table 4.

First, we checked the assumptions of multivariate analysis of variance (MANOVA). We found that the homogeneity assumption of the spread matrix (Box’s M) was met (F_(10–77,841,308)_ = 1631, Sig. = 0.091). When the Wilks’ Lambda test results are examined in Table 5, it is seen that the linear combinations of the sub-dimensions of the pre-service teachers’ technology addiction scale differed significantly in terms of gender variable (Wilks’ Λ = 0.887, F_(4,207)_ = 6604, Sig. = 0.000, ƞ^2^ = 0.113). According to this result, it differed between male and female pre-service teachers. The 0.113 eta squared indicated the results being close to a high level.

The examination aimed to identify significant differences among the dependent variables, and the results are shown in Table 6.

Table 6 shows the significant difference in playing online game sub-dimension according to gender variable (F_playing online game (1–210)_ = 19,280, Sig. = 0.000, ƞ^2^ = 0.084). The calculated effect size indicates a ‘moderate’ effect. That is to say, 8% of the variance of the scores obtained from the online game-playing sub-dimension can be explained by the gender variable. The mean scores show that male pre-service teachers (M = 11.70) engaged in online gaming more frequently than female pre-service teachers (M = 8.61).

The mean and standard deviation values of the technology addiction of the pre-service teachers regarding the gender variable are shown in Table 7.

When the table is examined (Table 7), it is observed that males were moderately technology addicted and females were low technology addicted, according to the ranges specified in the technology addiction scale.

The Pearson correlation coefficient results for the relationship between attitudes toward reading and technology addiction of pre-service teachers are shown in Table 8.

According to the analysis results (Table 8):

- There was a significant negative correlation between the love of reading book sub-dimension and online playing sub-dimension [r = −0.249, n = 212, Sig. < 0.001];
- There was a significant negative correlation between the benefits of reading books and using social network sub-dimension [r = −0.186, n = 212, Sig. < 0.001]; between instant messaging [r = −0.166, n = 212, *p* < 0.05] and playing online games [r = −0.318, n = 212, Sig. < 0.001], sub-dimensions, and a significant negative relationship between technology addiction total scores [r = −0.237, n = 212, Sig. < 0.001];
- There was a significant negative correlation between reluctance and stress sub-dimension in reading books and using social network [r= −0.191, n = 212, Sig.< 0.001]; and also, there was a significant negative relationship between instant messaging [r = −0.220, n = 212, Sig. < 0.001], using the website [r = −0.178, n = 212, Sig. < 0.001], sub-dimensions, and technology addiction total scores [r = −0.298, n = 212, *p* < 0.001].
- There was a significant negative correlation between attitude scores toward reading and using social network [r = −0.141, n = 212, Sig. < 0.05], instant messaging [r = −0.162, n = 212, Sig. < 0.05], playing online games [r = −0.371, n = 212, Sig. < 0.001], and technology addiction total scores [r = −0.246, n = 212, Sig. < 0.001].

## 4. Discussion

The attitude toward reading is a mental activity accompanied by emotions that increase or decrease students’ reading probability [43]. Language teachers are important in gaining reading habits [44,45,46]. However, more than just language teachers is needed to gain the habit of reading [46].

If pre-service teachers are unwilling to read or have free choices about reading, they will not be role models in instilling a love of reading in children [47]. Children need to see people reading books around them [48]. For this reason, teachers, whom students take as role models, should have good reading habits [49]. Similarly, Baccus [50] states that teachers’ experiences in reading habits and mentoring students have an important role in reading motivation. Therefore, teachers need to have a positive attitude in increasing students’ reading habits or increasing their reading motivation.

In this study, we concluded that pre-service teachers’ attitudes toward reading are positive and there are significant differences in terms of gender variable. We found a significant difference in favor of female pre-service teachers in the sub-dimensions of love of reading and reluctance and stress in reading books. Many studies examining attitudes toward reading books found significant differences in favor of female students [51,52,53]. Gömleksiz [54] and Özbay et al. [55] concluded that female students are likely to have more positive reading attitudes. Arslan et al. [56] and Biçer and Alan [57] also concluded that female pre-service teachers exhibit stronger reading habits than their male counterparts. Applegate and Applegate [44] found that pre-service teachers who enjoy reading have higher levels of success. Based on these results, we can conclude that female pre-service teachers’ reading habits are related to their success.

We found a significant difference between the gender variable of pre-service teachers and their technology addiction in the sub-dimension of playing online games. In the sub-dimension of playing online games, we found that male students play more online games than female students. Similar research findings to this result are also available. [58,59,60]. Our findings also indicate that males exhibit a moderate level of technology addiction, while females display a lower degree. Similar studies on technology addiction show men are more technology-addicted [61].

We found a negative significant correlation between technology addiction and attitude toward reading. Çakır and Akman [62] reached a similar conclusion due to their research. Researchers found a negative meaningful relationship between secondary school students’ digital game addiction and their reading habits. Fazla et al. [63] also found a negative relationship between high school students’ reading habits and smartphone addiction. Günlü and Oral [64] found a negative significant relationship between primary school students’ reading and writing attitudes and computer game addiction. Sağır and Eraslan [65] concluded in their research that students use digital tools very little for reading-related applications. In addition, they stated that with the increase in the use of smartphones and the effect of digitalization, young people are moving away from the reading culture. Levine et al. [66] also researched that decreased reading and the cessation of reading negatively affect academic life. They emphasized that this is because digital devices are associated with communication. They also saw this problem as a global problem. The results in the literature show that students’ reading habits and performance are negatively affected by spending too much time on social media and that it causes distraction [67,68,69]. Ghufron et al. [70] concluded that smartphone-addicted students have lower psychological well-being. Alhrahsheh and Almajali [71] also found a negative relationship between social network addiction and students’ academic performance in their study.

The hypotheses determined in the research were accepted. Similar studies also support finding a significant relationship between technology addiction and reading attitude. The more technology addictions of pre-service teachers increase, the lower their reading attitudes will decrease.

## 5. Conclusions

The present study, which aimed to examine the attitudes of pre-service teachers toward reading books and their technology addiction, revealed that while pre-service teachers tend to have positive attitudes towards reading books, they also tend to exhibit divergent attitudes depending on their gender. The inquiry about technology addiction of the pre-service teachers depending on the gender variable revealed a significant difference in the “playing online game sub-dimension”. In the playing online game sub-dimension, it is understood that male students play more online games than females. There is a negative relationship between technology addiction and attitude towards reading. This result indicates that an increased attitude towards reading books reduces technology dependency.

Based on the results obtained from the research, further research can be suggested to increase the attitudes of male pre-service teachers towards reading books and reduce males’ technology addiction. It may also be suggested to conduct causal–comparative research to increase attitudes towards reading books and to reduce technology addiction.

In addition, teachers should be role models for their students by improving their technological competencies, which are among the 21st-century skills. For this reason, it is expected for all teachers to decrease their technology addiction by improving their technological competencies and also to develop positive attitudes toward reading books.

## 6. Limitations

This research had some limitations. First and foremost, since the research employed a quantitative methodology, its scope was confined to the data collected solely from the scales. Therefore, researchers are encouraged to examine pre-service teachers’ attitudes toward reading books and their technology addiction in depth. Second, this research was limited to the sample for which data were collected. Further studies making use of larger sample groups can be conducted.

## Figures and Tables

**Table 1:** Descriptive statistical results of book reading attitude according to gender.

Sub Dimensions	Gender	$\bar{X}$	sd
Love for Reading	Female	45.23	8.59
	Male	39.17	10.99
Total		43.35	9.79
Benefits of Reading	Female	35.80	5.50
	Male	33.95	6.10
Total		35.22	5.75
Reluctance and Stress in Reading	Female	38.87	3.57
	Male	37.40	4.05
Total		38.41	3.78
Obstacles in Reading	Female	21.03	2.56
	Male	21.49	2.32
Total		21.18	2.49
Total	Female	140.79	15.43
	Male	132.02	18.01

**Table 2:** MANOVA analysis results regarding the difference between the average of pre-service teachers’ attitudes toward reading books according to gender variable.

	Wilks’ Λ	F	Hypothesis df	Error df	Sig.	ƞ^2^
Gender	0.903	5.549	4	207	0.000	0.097

**Table 3:** Results regarding the difference between pre-service teachers’ reading book attitude sub-dimensions according to gender variable.

Source of Variance	Dependent Variable	Sum of Squares	df	Mean Square	F	Sig. *	ƞ^2^
Gender	“Love for Reading”	1669.529	1	1669.529	18.868	0.000	0.082
	“Benefits of Reading”	155.216	1	155.216	4.785	0.030	0.022
	“Reluctance and Stress in Reading”	97.215	1	97.215	7.011	0.009	0.032
	“Obstacles in Reading”	9.657	1	9.657	1.564	0.212	0.007
Error	“Love for Reading”	18,581.713	210	88.484			
	“Benefits of Reading”	6811.441	210	32.435			
	“Reluctance and Stress in Reading”	2911.788	210	13.866			
	“Obstacles in Reading”	1296.380	210	6.173			
Total	“Love for Reading”	418,559.479	212				
	“Benefits of Reading”	269,985.064	212				
	“Reluctance and Stress in Reading”	315,802.942	212				
	“Obstacles in Reading”	96,384.548	212				

* The new significance level obtained according to the Bonferroni correction is 0.012.

**Table 4:** Descriptive statistics results of the pre-service teachers’ technology addiction on the gender variable.

Sub Dimensions	Gender	$\bar{X}$	sd
Using Social Network	Female	12.42	4.94
	Male	12.69	4.23
Total		12.50	4.72
Instant Messaging	Female	13.59	4.99
	Male	13.48	5.09
Total		13.55	5.01
Playing Online Game	Female	8.61	4.48
	Male	11.69	5.26
Total		9.57	4.93
Using Websites	Female	12.95	5.60
	Male	14.00	4.95
Total		13.28	5.42

The results of the MANOVA analysis regarding the difference between gender and technology addiction are shown in Table 5.

**Table 5:** The results of MANOVA analysis on the difference between the technology addiction score averages of pre-service teachers according to gender variable.

	Wilks’ Λ	F	Hypothesis df	Error df	Sig.	ƞ^2^
Gender	0.887	6.604	4	207	0.000	0.113

**Table 6:** Results regarding the difference between the technology addiction sub-dimensions of pre-service teachers according to gender variable.

Source of Variance	Dependent Variable	Sum of Squares	df	Mean Square	F	Sig. *	ƞ^2^
“Gender”	“Using Social Network”	3.464	1	3.464	0.155	0.695	0.001
	“Instant Messaging”	0.532	1	0.532	0.021	0.885	0.000
	“Playing Online Game”	432.283	1	432.283	19.280	0.000	0.084
	“Using Websites”	50.019	1	50.019	1.711	0.192	0.008
Error	“Using Social Network”	4703.098	210	22.396			
	“Instant Messaging”	5289.720	210	25.189			
	“Playing Online Game”	4708.557	210	22.422			
	“Using Websites”	6137.465	210	29.226			
Total	“Using Social Network”	37,867.946	212				
	“Instant Messaging”	44,258.710	212				
	“Playing Online Game”	24,571.189	212				
	“Using Websites”	43,565.833	212				

* The new significance level obtained according to the Bonferroni correction is 0.012.

**Table 7:** Distribution of technology addiction by gender.

Gender	n	$\bar{X}$	sd	Comment
Female	146	47.58	16.50	Low addicted
Male	66	51.88	15.83	Moderately addicted

**Table 8:** Relationship analysis between book reading attitude and technology addiction.

Pearson Correlation		Using Social Network	Instant Messaging	Playing Online Game	Using Websites	Technology Addiction
Love for Reading	r	−0.026	−0.055	−0.249 **	−0.079	−0.125
Benefits of Reading	r	−0.186 **	−0.166 *	−0.318 **	−0.111	−0.237 **
Reluctance and Stress in Reading	r	−0.191 **	−0.220 **	−0.390	−0.178 **	−0.298 **
Obstacles in Reading	r	−0.034	−0.077	−0.068	0.007	−0.052
Attitude toward Reading	r	−0.141 *	−0.162 *	−0.371 **	−0.133	−0.246 **

n:212, ** Sig. < 0.01, * Sig. < 0.05.

## Data Availability

Data will be made available upon request.
